# Antibiotic resistance, virulence, and phylogenetic analysis of *Escherichia coli* strains isolated from free-living birds in human habitats

**DOI:** 10.1371/journal.pone.0262236

**Published:** 2022-01-12

**Authors:** Bartosz Rybak, Beata Krawczyk, Beata Furmanek-Blaszk, Magdalena Wysocka, Magdalena Fordon, Pawel Ziolkowski, Wlodzimierz Meissner, Katarzyna Stepniewska, Katarzyna Sikorska

**Affiliations:** 1 Department of Environmental Toxicology, Faculty of Health Sciences with Institute of Maritime and Tropical Medicine, Medical University of Gdańsk, Gdańsk, Poland; 2 Department of Molecular Biotechnology and Microbiology, Faculty of Chemistry, Gdańsk University of Technology, Gdańsk, Poland; 3 Department of Microbiology, Faculty of Biology, University of Gdańsk, Gdańsk, Poland; 4 Avian Ecophysiology Unit, Department of Vertebrate Ecology and Zoology, University of Gdańsk, Gdańsk, Poland; 5 Department of Tropical Medicine and Parasitology—National Centre for Tropical Medicine, Faculty of Health Science with Institute of Maritime and Tropical Medicine, Medical University of Gdańsk, Gdynia, Poland; Maria Curie-Sklodowska University, POLAND

## Abstract

Wild birds can be colonized by bacteria, which are often resistant to antibiotics and have various virulence profiles. The aim of this study was to analyze antibiotic resistance mechanisms and virulence profiles in relation to the phylogenetic group of *E*. *coli* strains that were isolated from the GI tract of wildfowl. Out of 241 faecal samples, presence of *E*. *coli* resistant to a cephalosporin (ESBL/AmpC) was estimated for 33 isolates (13,7%). Based on the analysis of the coexistence of 4 genes encoding ESBLs/AmpC (*bla*_CTX-M_, *bla*_TEM,_ *bla*_SHV_, *bla*_AmpC_) and class 1 and 2 integrons genes (*int*I1, *int*I2) a subset of two resistance profiles was observed among the investigated *E*. *coli* isolates carrying *bla*_AmpC_, *bla*_SHV_, and *bla*_CTX-M_, *bla*_TEM_, class 1 and 2 integrons, respectively. The *E*. *coli* isolates were categorized into 4 phylogenetic groups A (39.4%), B2 (24.25%), D (24.25%) and B1 (12.1%). The pathogenic B2 and D groups were mainly typical for the Laridae family. Among the 28 virulence factors (Vfs) detected in pathogenic phylogenetic groups B2 and D, 7 were exclusively found in those groups (*sfa*, *vat*, *tosA*, *tosB*, *hly*, *usp*, *cnf*), while 4 VFs (*fecA*, *fyuA*, *irp2*, *kspMTII*) showed a statistically significant association (P≤0.05) with phylogroups A and B1. Our results indicated that strains belonging to commensal phylogroups A/B1 possess extensive iron acquisition systems (93,9%) and autotransporters (60,6%), typical for pathogens, hence we suggest that these strains evolve towards higher levels of virulence. This study, which is a point assessment of the virulence and drug resistance potential of wild birds, confirms the importance of taking wild birds as a reservoir of strains that pose a growing threat to humans. The *E*. *coli* analyzed in our study derive from different phylogenetic groups and possess an arsenal of antibiotic resistance genes and virulence factors that contribute to their ability to cause diseases.

## Introduction

Dissemination of multidrug-resistant (MDR) bacteria is a significant worldwide problem [1]. Antibiotic resistance is the result of the ability of microorganisms to mutate, acquire mobile genetic elements encoding resistance genes and to transfer plasmids between hosts. The production of extended-spectrum β-lactamases (ESBLs), AmpC-type β-lactamases and carbapenemases is one of the most clinically and epidemiologically important mechanisms of resistance to antibiotics in *Enterobacterales* [2]. At the present time, the most prevalent ESBLs in Europe and in other areas of the world are cefotaximase-Munich (CTX-M), Temoneira β-lactamase (TEM) and sulfhydryl variant (SHV-type) ESBLs. These traits are mainly detected in hospital strains, although recently they have also been more frequently found in community-acquired strains [3]. *E*. *coli* is most commonly used as a representative indicator of antimicrobial resistance in Gram-negative bacteria and may be relevant to human as well as veterinary medicine [2]. A worldwide increase in ESBL-producing *E*. *coli* isolates in both community and hospital settings has been observed since early 2000 [3]. The transmission of *E*. *coli* between hosts begins from gut colonisation. On the basis of virulence factors *E*. *coli* can be classified into intestinal pathogenic (IPEC) and extraintestinal pathogenic (ExPEC) strains. The ExPEC pathotype can be further subdivided into uropathogenic *E*. *coli* (UPEC), neonatal *E*. *coli* meningitis (NMEC), *E*. *coli* associated with sepsis (SEPEC) and avian pathogenic *E*. *coli* (APEC). Whereas IPEC strains are obligate pathogens, ExPEC are facultative pathogens which belong to the normal gut flora and do not provoke the symptom of disease. However, a transition from asymptomatic colonisation of the gut to the urinary tract or other organs outside of the digestive system, causing infection, can happen. ExPECs probably do not exhibit host specificity or it is wide, including humans and birds [4].

Apart from phenotypic and molecular analysis of antibiotic resistance mechanisms, virulence profiles of bacteria are one of the most important issues in recent microbiological studies [5–7]. A broad spectrum of virulence factors (VFs) mediates *E*. *coli* pathogenesis. These factors can be specific to enteric or to ExPEC pathogens. *E*. *coli* virulence is mediated by adhesion, toxins production, polysaccharide capsules production, iron acquisition (siderophores), invasins, and production of other factors targeting immune cells. Such VFs help to colonize the surfaces of host cells, avoid and/or abolish the host defense mechanisms, damage and/or enter host cells or tissues, including blood components, and provoke a harmful immune response, which increases the risk of disease [1]. Avian and human *E*. *coli* isolates carry a similar set of genes encoding VFs, in addition to this, they often belong to the same phylogenetic groups. It may indicate the zoonotic origin of ExPEC [8]. Each of the VF contributes to the fitness of bacteria but siderophore-mediated iron uptake systems encoded in the bacterial cell give a fully virulent phenotype. Iron is an essential trace element for most bacteria and the presence of multiple iron uptake systems facilitates *E*. *coli* gut colonisation and also plays an important role in extraintestinal virulence [9]. Studies conducted on APEC and UPEC have shown that both groups of strains utilize similar iron acquisition mechanisms, especially salmochelin and aerobactin systems [10]. It is suggested that the pathogenicity of ExPEC results not only in toxins but also siderophores production [11, 12].

According to Clermont et al. *E*. *coli* strains can be categorized into seven *sensu stricto* phylogenetic groups: A, B1, B2, C, D, E, F, while the eighth group is called a cryptic *Escherichia* clade I [13–15]. VFs play an important role in this classification. Commensal *E*. *coli* colonize the gastrointestinal tract mucosa and most often represent groups A or B1. The pathogenic strains responsible for intestinal infections are represented by A, B1 and group D, while most ExPEC strains belong to phylogenetic group B2 followed by group D [16–19].

The relationship between humans and farm animals has already been proven many times, but there are still only a few reports on the association of infections in humans with a potential source among wild birds[20–23]. Meanwhile, nowadays, the assessment of the amount of various anthropogenic pollutants entering the environment is of key importance. Wild birds, especially waterfowl are often taken into account as indicators of ecosystem health, reflecting changes in habitats, increased disease incidence, and exposure and effects of biological and chemical pollution [24–27]. Moreover, migratory birds can transport microorganisms to distant sites where different bird species often congregate in large numbers, i.e. at stopover sites or wintering areas. In such places, horizontal transmission might occur, especially among individuals residing in water bodies that host dense flocks, which waterfowl often do [26, 28]. Hence, migrating wild birds can play an important epidemiological role in spreading bacteria resistant to antibiotics in the environment which could potentially become a hazard to human and animal health [29–33].

There is little known about the occurrence of drug-resistant bacteria in wild birds living in Poland as, so far, no extensive research has been carried out on this topic. Hence, the aim of this study was to determine the prevalence of ESBL/AmpC *E*. *coli* strains in wild birds living close to human habitats in northern and central part of Poland. *E*. *coli* strains isolated from the gastrointestinal tract (GI) of wild-fowl were screened for their antibiotic resistance mechanisms and virulence profiles.

## Materials and methods

### Sampling and collection of *E*. *coli* avian strains

The bird faeces samples were collected from the cloaca of 241 birds belonging to the Anatidae families (Mallard *Anas platyrhynchos*—128, Mandarin Duck *Aix galericulata*– 1, Mute Swan *Cygnus olor* -5), Laridae (Black-headed Gull *Chroicocephalus ridibundus* -38, Common Gull *Larus canus* -12, European Herring Gull *Larus argentatus* -16), Rallidae (Eurasian Coot *Fulica atra-*29, Common Moorhen *Gallinula chloropus* -1) and Corvidae (Hooded Crow *Corvus cornix*—5, Jackdaw *Corvus monedula*—4, Rook *Corvus frugilegus*– 2) between January 2017 and October 2018 (S1 Table). The birds were sampled in northern and central Poland on different urban water reservoirs and on municipal beaches of the Baltic Sea Coast. All the bird handling and sample collecting was performed by persons with a valid ringing license, issued by the Ornithological Station of the Polish Academy of Sciences approved by the General Directorate for Environmental Protection in Poland (approval number: DZP-WG.6401.102.2020.TL) and the Ministry of the Environment (approval number: DL-III.6713.11.2018.ABR). Cloacal swabs were collected during the routine procedure of bird ringing without any unnecessary harm to the animals. After the sampling, the birds were immediately released. After the collection, the faecal samples were immersed in Amies transport medium and transported in an isothermal box to the laboratory. Within 3–4 h, the samples were analyzed for the presence of ESBL/AmpC-producing isolates with the use of cefotaxime-supplemented MacConkey agar (MCA) medium (2 mg/l). If a sample scored positive for the presence of antibiotic resistant bacteria, one lactose positive colony grown on MCA was selected, grown to pure culture and further analyzed. Isolated antibiotic-resistant coliform colonies were identified using MALDI-TOF MS (MALDI biotyper; Bruker Daltonics, USA) according to the manufacturer’s instructions. After that, only the strains identified as *E*. *coli* were used for further analysis (n = 33). The purified isolates were grown on LA (Graso, Poland) medium and then stored at -80°C in 20% glycerol stocks.

### Antimicrobial susceptibility testing

Cefotaxime-resistant *E*. *coli* isolates were tested using the double-disk synergy (DDS) test to identify ESBL-producing strains. The DDS assay was carried out according to European Committee for Antimicrobial Susceptibility Testing (EUCAST) v.10.0 (2020) guidelines [34]. 33 isolates showing reduced susceptibility to cefotaxime were tested for susceptibility to the following 16 antimicrobial pharmaceuticals (Oxoid): ampicillin (10 μg), amoxicillin/clavulanic acid (10/30 μg), piperacillin/tazobactam (30/6 μg), cefuroxime (30 μg), ceftazidime (10 μg), cefepime (30 μg), cefotaxime (5 μg), ciprofloxacin (5 μg), amikacin (10 μg), gentamicin (10 μg), netylmicin (10 μg), meropenem (10 μg), imipenem (10 μg), ertapenem (10 μg), sulfamethoxazole/trimethoprim (1.25/23.75 μg) and tigecycline (15 μg). A reference strain of *E*. *coli* ATCC 25922 was used as a control.

### Bacterial DNA isolation

The genomic DNA of bacterial isolates (n = 33) was purified using the GENOMIC DNA KIT (BLIRT S.A., Gdansk, Poland) according to the manufacturer’s protocol. The concentration of extracted DNA was measured by NanoDrop ND-100 (Thermo Fisher Scientific, Wilmington, USA). For the detection of the plasmid-born genes, DNA was isolated from bacterial strains by standard alkaline lysis and ethanol precipitation [35].

### Antibiotic resistance genes and integrons detection

The presence of gene coding for antimicrobial resistance mechanisms was verified in ESBL/AmpC-positive strains using molecular methods. Simplex polymerase chain reaction (PCR) was used for the detection of the ESBL gene’s phenotype (*bla*_CTX-M_, *bla*_SHV_, *bla*_TEM_), and AmpC β–lactamase gene (*ampC*). The presence of integrons was detected by PCR amplification of the integrase gene *intI1* (for class 1 integrons) and *intI2* (for class 2 integrons). Amplicons of *bla*_CTX-M_ were randomly selected for sequencing (Genomed S.A. Warsaw, Poland). Specific primers and the PCR conditions were as described previously [36, 37].

### *E*. *coli* molecular typing

The polymerase chain reaction melting profiles (PCR MP) procedure was used to assess the genomic diversity of the isolated *E*. *coli*. The PCR MP was carried out as described in Krawczyk et al. [38]. Amplified genomic DNA fragments were separated using polyacrylamide gel electrophoresis (6%) in 1×TBE buffer and stained with ethidium bromide. Images of the gels were analyzed and archived using the Versa Doc Imaging System version 1000 (Bio-Rad Laboratories, Hercules, USA). Dendrogram was generated by the Dice Coefficient (DC) with UPGMA method and with a setting of 1% band tolerance (FPQuest TM software, BioRad; version 4.5).

### *E*. *coli* virulence genes detection

The detection of 31 genes associated with bacterial virulence patterns was performed. The genes included those coding for fimbrial and afimbrial family adhesins Afa/Dr (*afa/dr*), fimbriae: 1 (*fimH*), 3 (*mrkD*), P (*papG*), S (*sfaD/sfaE*), F1C (*focG)*, toxins: α-hemolysin (*hlyA*), uropathogenic specific protein (*usp*), cytotoxic necrotizing factor 1 (*cnf1*), siderophores: enterobactin (*iha*), yersiniabactin receptor (*fyuA*), aerobactin receptor (*iutA*); invasin A (*ibeA*); gene of capsule synthesis (*kspMTII*); and autotransporter facilitating biofilm formation (*ag43*). The presence of genes was verified with multiplex or simplex PCR as described previously [16, 22, 23, 39]. Nine genes coding for the ABC-transporter protein (*tosB*), toxin TosA (*tosA)*, enterobactin synthesis (*entB*), salmochelin receptor (*iroN)*, enterobactin receptor (*fepA*), ferric citrate outer membrane transporter (*fecA*), yersiniabactin synthesis (*irp2*), aerobactin synthesis (*iucA*) and autotransporter (*aida*) were detected as described in Krawczyk et al. [40]. The presence of serine protease autotransporters (SPATE) genes *sat*, *vat*, *pic*, *pic*-like (U), *boa*, *hbp* and *pssA* were detected with polymerase chain reaction/restriction fragment length polymorphism (PCR/RFLP) (HaeIII) as previously described [41]. For PCR analysis, the uropathogenic reference *E*. *coli* strain CFT073 (ATCC 700928) and clinical *E*. *coli* strains from the collection of the Gdansk University of Technology were used as the positive controls.

### Phylogenetic analysis

The phylogenetic group of each *E*. *coli* strain was determined according to the method developed by Clermont et al [15], by multiplex PCR of the genes *chuA*, *yjaA* and the DNA fragment TSPE4.C2.

### Statistical analysis

Comparisons of the relative proportions of virulence genes were tested using Fisher’s exact test or Pearson’s χ2 test. Fisher’s exact test was used to compare frequencies between groups. The threshold for statistical significance was a P value <0.05. The virulence scores of the strains are reported as the sum of the virulence genes they possessed. The Mann–Whitney U test was used to compare virulence scores among groups. Cluster analysis was based on the presence and absence of virulence genes and resistance genes. A binary matrix was used to determine similarities using the Euclidean distance and complete linkage, and strains were grouped using R package *stats* [42]. Dendrograms were constructed using the packages *ggplot2* [43] and *ggdendro* [44] R version 3.5.3.

## Results

### ESBL/AmpC–producing *E*. *coli* strains detection

Over the past decades multi-resistant ESBL-producing *E*. *coli* have become a prototype species for the spread of antimicrobial resistance into wildlife [45]. A total of 241 faecal samples from cloacal swabs of wild birds were screened for reduced sensitivity to third-generation cephalosporins. Of these 52 (21.5%) strains showed ESBL/AmpC phenotype and 33 isolates were confirmed as *E*. *coli*. These 33 strains were recovered from birds belonging to 6 different avian species (11 Mallards; 4 Black-headed Gull; 3 European Herring Gulls; 4 Common Gulls; 10 Eurasian Coots; 1 Jackdaw) (S1 Table).

The results showed, there is a statistically significant (*P* = 0.005) difference between four of the studied families in gut colonisation with ESBL/AmpC strains (Table 1).

**Table 1:** Frequency (%) of ESBL/AmpC *E coli* strains among four bird families.

Family	Anatidae (n = 134)	Laridae (n = 66)	Corvidae (n = 11)	Rallidae (n = 30)	*P* value^1^
ESBL/AmpC *E*. *coli*	11 (8.2)	11 (16.7)	1 (9.1)	10 (33.3)	**0.005**

^1^ Comparison of frequencies among different groups was done by the Fisher exact test. The threshold for statistical significance was a *P* value <0.05.

### Antibiotic susceptibility of *E*. *coli* from avian GI-tract

Antibiotic sensitivity/resistance data obtained after standard disc diffusion tests showed the highest resistance towards a β-lactam and β-lactamase-inhibitor combination: ampicillin and cefuroxime (100%), cefotaxime and ceftriaxone (97%), amoxicillin/clavulanic acid (91%), ceftazidime (88%), cefepime (70%), and piperacillin/tazobactam (61%), followed by sulphamethoxazole/trimethoprim resistance which was 76%. The percentage of aminoglycosides (amikacin, gentamicin and netilmicin) resistant *E*. *coli* was 27%, 24% and 24%, respectively. The high degree of resistance was observed for tigecycline (48%) and ciprofloxacin (45%). All of the ESBL/AmpC producing strains were susceptible to ertapenem, imipenem and meropenem. The majority of *E*. *coli* strains of our research showed a high prevalence of resistance against commonly prescribed first-line antibiotics.

### Distribution of genes coding for ESBL resistance, AmpC lactamase and integrons class 1 and 2 among *E*. *coli* isolated from birds

In *Enterobacterales* and other Gram-negative bacteria, ESBLs often possess one of three types of genes: *bla*_CTX-M_, *bla*_TEM_ and *bla*_SHV_, which enable them to mediate resistance to β-lactams. In this study, we confirmed that the majority of isolated *E*. *coli* strains (29 out of 33) (87.9%) were found to possess genes coding for CTX-M, conferring a high level of resistance to oxyimino-β-lactams, especially to cefotaxime, ceftriaxone and cefuroxime. DNA sequencing of 10 randomly chosen PCR products revealed sequence identity with *bla*_CTX-M-15_ (GenBank Accession No. AY044436). 25 ESBL-positive isolates carried the gene encoding the TEM β-lactamase, while the *bla*_SHV_ gene was only identified in 6 isolates. Coexistence of ESBL and AmpC β-lactamases was detected in eight (24%) isolates.

A high percentage of ESBL/AmpC-producing isolates in the current study harbored integrons. 72.7% of *E*. *coli* strains carried the integron-integrase 1 gene (*intI1*), while 51.5% of the isolates carried the integron-integrase 2 gene (*intI2*). Among the 33 confirmed ESBL/AmpC producers, 23 (69.7%), 20 (60.6%) and 4 (12%) showed the presence of *bla*_CTX-M_, *bla*_TEM_, *bla*_SHV_ and *intI1*, respectively. Among the 33 confirmed ESBL/AmpC producers, 17 (51.5%), 17 (51.5%) and 3 (9%) showed the presence of *bla*_CTX-M_, *bla*_TEM_, *bla*_SHV_ and *intI2*, respectively. The most common combination of ESBL genes (*bla*_CTX-M_, and *bla*_TEM_) together with class 1 and 2 integrons was observed in 9 strains (27%). The distribution of the genes encoding the ESBL phenotype, *ampC* gene and integrons of class 1 and 2 is presented in Table 2.

**Table 2:** Distribution of ESBLs encoding genes (*bla*_CTX-M_, *bla*_TEM_ and *bla*_SHV)_, AmpC β–lactamase gene (*ampC*) and class 1 and 2 integrons (*intI1*, *intI2)* among phenotypic-positive ESBL/AmpC producers (n = 33) isolated from wild birds.

Genotype	Number (%)
CTX-M + TEM + AmpC + Int1 + Int2	2 (6)
CTX-M + TEM + SHV + Int1 + Int2	3 (9)
CTX-M + TEM + SHV + Int1	1 (3)
**CTX-M + TEM + Int1 + Int2**	**9 (27)**
CTX-M + TEM + Int1	4 (12)
CTX-M + TEM + Int2	3 (9)
CTX-M + TEM + AmpC	1 (3)
CTX-M + AmpC + Int1	3 (9)
CTX-M + Int1	1 (3)
TEM + AmpC + Int1	1 (3)
TEM + AmpC	1 (3)
CTX-M	2 (6)
SHV	2 (6)

**Fig 1:**
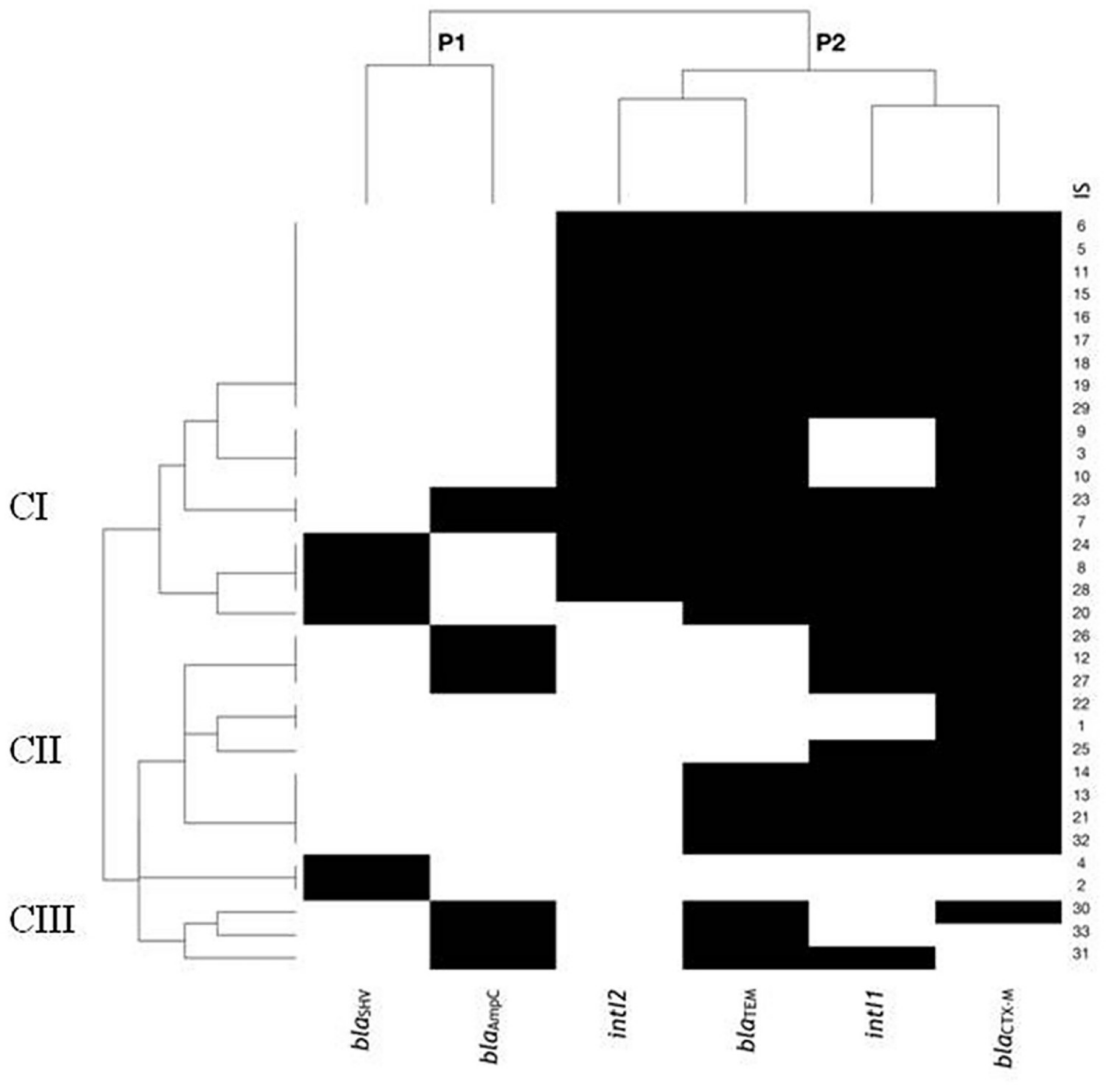
Dendrogram showing relationship between *E*. *coli* strains (n = 33) by ESBLs and AmpC encoding genes and profile of class 1 and 2 integrons (on the left). The top of the dendrogram shows the relationship between genes. Black spots represent the presence and white spots represent the absence of genes. IS–isolates number; CI, CII, CIII–clades: I, II, III. Description of the graph in the text.

Based on an analysis of the coexistence of six genes encoding ESBLs/AmpC resistance and class 1 and 2 integrons, the relationship between the strains was estimated (Fig 1).

A subset of two different patterns of genes (P1, P2) was observed among the investigated *E*. *coli* isolates. Profile P1 grouped together *bla*_AmpC_ and *bla*_SHV_ genes was mainly associated with clade I. The second profile (P2) included *bla*_CTX-M_ and *bla*_TEM_ genes, as well as both the class 1 and class 2 integrase genes, and was also more common in clade I. We also observed the complete absence of class 2 integrons in clade II and III. This finding suggests that the resistance profile of the bacterial isolates in this study may not only be linked to integrons.

### Avian ESBL/AmpC–producing *E*. *coli* belong to diverse phylogenetic groups

All isolates were grouped to phylogenetic clades according to Clermont et al. [15]. The *E*. *coli* isolates belonged to the phylogenetic groups A (39.4%), B2 (24.25%), D (24.25%) and B1 (12.1%). Phylogenetic typing demonstrated that strains isolated from the Anatidae were mainly classified in A phylogroup (64%), isolates from the Rallidae were classified in the B2 (50%) or A phylogroup (40%), while strains isolated from Laridae belonged to all phylogroups (Table 3). Only one ESBL/AmpC *E*. *coli* strain isolated from Corvidae was assigned to group D. The ESBL/AmpC *E*. *coli* strains that belonged to phylogroup B2 were isolated from only two families (Laridae, Rallidae), which is statistically significant (P = 0.041).

**Table 3:** Prevalence of phylogenetic groups among ESBL/AmpC-positive *E*. *coli* according to bird families.

Family / Phylogenetic groups	Anatidae (n = 11)	Laridae (n = 11)	Corvidae (n = 1)	Rallidae (n = 10)	*P* value^1^
A (n = 13)	7	2	0	4	0.124
B1 (n = 4)	1	2	0	1	1.0
**B2** (n = 8)	0	3	0	5	**0.041**
D (n = 8)	3	4	1	0	0.063

^1^ Comparison of frequencies among different groups was done with the Fisher exact test (*P* value <0.05).

### Distribution of ESBLs encoding genes according to phylogenetic groups

**Fig 2:**
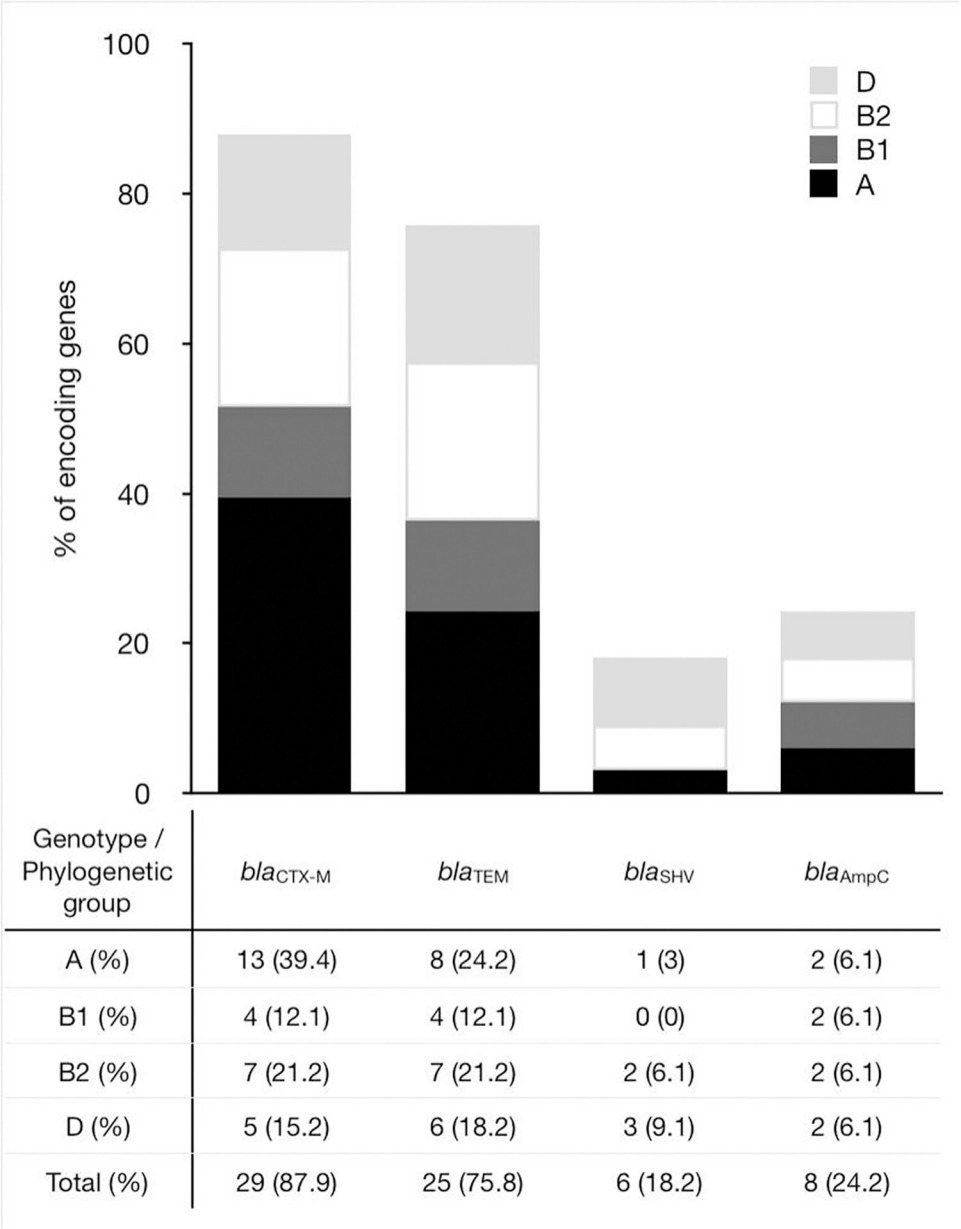
Distribution of ESBLs and AmpC encoding genes among phenotypic-positive ESBL/AmpC producers (n = 33) isolated from birds according to phylogenetic groups.

The occurrence and coexistence of *bla*_CTX-M_, *bla*_TEM_, *bla*_SHV_ and *bla*_AmpC_ genes in various phylogenetic groups (A, B1, B2 and D) were analyzed (Fig 2).

The *bla*_CTX-M_ gene, similarly to *bla*_TEM_, appeared most often in the commensal group A (39.4% and 24.2%, respectively). Their co-occurrence (*bla*_CTX-M_ + *bla*_TEM_) in this group was also the most often recorded (24.2%). The *bla*_CTX-M_ + *bla*_TEM_ gene combination was equally distributed (12.1%) among phylogroups B1, B2 and D. The other β-lactamase gene, *bla*_SHV,_ was more frequently observed in the group of B2 (6.1%) and D (9.1%) pathogenic strains. Only 3% of the strains carrying the *bla*_SHV_ gene were assigned to group A, while the *bl*a_SHV_ gene was not found in group B1. The coexistence of three *bla*_CTX-M_ + *bla*_TEM_ + *bla*_SHV_ genes was identified in 4 isolates belonging to the B2 (6.1%), A and D (3% each) phylogroups. However, no such gene set was found in group B1. Our research showed that the phylogenetic group B2 is the richest in genes associated with ESBL resistance.

### Virulence factors (VFs) and their prevalence in phylogenetic groups

Genes encoding the VFs associated with the production of adhesins, siderophores and other iron uptake systems, toxins, autotransporters and genes associated with capsule and biofilm synthesis were sought. The most frequent virulence genes such as: *fepA* (siderophore uptake transmembrane transporter activity), *entB* (enterobactin synthase component B), *fimH* (type 1 fimbriae) and *fecA* (the outer membrane receptor), were observed in ≥60% of the isolates. In contrast, 13 genes, including *usp* (uropathogenic specific protein), *iha* (putative adhesin-siderophore) and *papG* (P fimbriae), were present in less than 10% of the isolates, whereas 5 virulence genes (*afa/Dr*, *ibeA*, *focG*, *pssA*, *boa*) were not detected among the 33 isolates. The median virulence score was 7 (range 1–16) for all isolates. Taking into account the division into commensal and pathogenic strains (according to the criteria of Clermont et al. 2007) [15] it was observed that out of the 31 examined virulence genes only *fecA* gene was significantly associated with the commensal group of isolates (A, B1) while, three (*kspMTII*, *irp2*, *fyuA*) were significantly associated with the pathogenic group of isolates (B2, D) (S2 Table). Virulence genotypes in relation to the individual phylogenetic group among 33 avian *E*. *coli* isolates were also analyzed. The median virulence score was higher for D and B2 groups (8 VFs (range 5–11) and 6.5 VFs (range 4–16), respectively) than VFs detected among strains from phylogroups A and B1 (6 VFs (range 1–14) and 5.5 VFs (range 4–12), respectively). It is worth noting that, among the 28 VFs detected in phylogroup B2, six were exclusively detected in that group (*vat*, *tosA*, *tosB*, *hly*, *usp* and *sfa*). Five VFs (*fecA*, *fyuA*, *aida*, *irp2*, *kspMTII*) showed a statistically significant association (P≤0.05) with phylogenetic groups A, B1, B2 and D. In Table 4 only statistically significant results are presented.

**Table 4:** Prevalence of virulence genes among *E*. *coli* isolates depending on phylogroup. The table presents only statistically significant results.

Category	Gene^1^	Gene prevalence (% of isolates)					*P value* ^2^
		All isolates (n = 33)	PhG-A isolates (n = 13)	PhG-B1 isolates (n = 4)	PhG-B2 isolates (n = 8)	PhG-D isolates (n = 8)	
Iron acquisition	*fecA*	22 (66.7)	12 (92.3)	3 (75)	4 (50)	3 (37.5)	0.034
	*fyuA*	18 (54.5)	3 (23)	2 (50)	6 (75)	7 (87.5)	0.015
	*irp2*	18 (54.5)	3 (23)	2 (50)	6 (75)	7 (87.5)	0.015
Autotransporters	*aida*	19 (57.6)	8 (61.5)	3 (75)	0 (0)	8 (100)	<0.001
Capsule related	*kspMTII*	6 (18)	0 (0)	0 (0)	2 (25)	4 (50)	0.015

^1^function of genes: *fecA*, ferric citrate outher membranę transporter; *fyuA*, yersiniabactin receptor; *irp2*, yersiniabactin gene synthesis; *aida*, autotransporter; *kspMTII*, synthesis capsule. PhG-phylogroup.

^2^ The prevalence rates of the virulence genes among commensal versus pathogenic isolates were compared by the Chi-squared test of independence or Fisher’s exact test. P values are shown only when P was <0.05.

### Phylogenetic analysis based on the virulence-associated gene (VAG) profile

**Fig 3:**
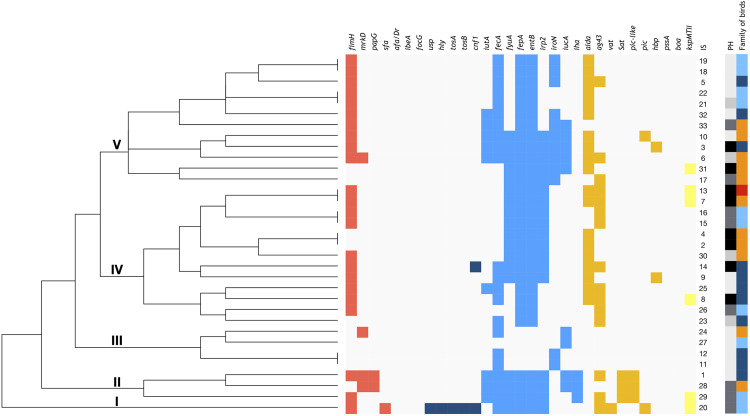
Dendrogram showing relationship between *E*. *coli* strains by virulence-associated gene (VAG) profile. Legend: At the top virulence genes are depicted, on the right the isolate number. Red spots represent adherence factors, deep blue spots represent toxins, blue spots represent iron uptake systems, orange spots represent autotransporters, yellow spots represent the capsule synthesis gene and white spots represent the absence of genes. IS**—**ID of strains (1–33) represent cluster designations. Category: PH (phylogenetic group): white spots represent A, light grey spots represent B1, dark grey spots represent B2, black spots represent D phylogroups; Family of birds: light blue spots represent the Rallidae bird family, deep blue spots Anatidae, orange spots Laridae and red spots Corvidae. I, II, III, IV and V–represent the clades created based on virulence genes. Cluster analysis was based on the presence and absence of virulence genes using R packages: *stats*, *ggplot2* and *ggdendro*, R version 3.5.3.

On the basis of virulence genes presence, the relationship between *E*. *coli* strains was assessed. On the cluster analysis, strains were distributed into five groups (Fig 3).

Group I is only represented by a single isolate (IS 20) assigned to the phylogenetic group B2, which possesses most of the virulence genes. This isolate is the only one that has the *sfa* gene encoding S- fimbriae and a set of genes typical for uropathogenic strains (*usp*, *hly*, *cnf1*, *tosA* and *tosB*). This strain was isolated from a sample of birds representing the Rallidae family. Group III has the least VFs. These isolates are assigned to phylogenetic group A and considered as a group of commensal strains. Groups IV and V have a comparable amount of VFs but are phylogenetically diverse. The phylogenetic groups D and B2 were predominant in Group IV. There were only 3 isolates in Group II and they have more virulence genes than isolates from Groups IV and V, but less than the isolate from Group I. We found that *fyuA*, *irp*2 and *kspMTII* virulence genes are significant in differentiation between the pathogenic strains and the commensals. The results of this study also show that all strains, in general, had at least one virulence gene related to iron acquisition. Furthermore, the strains belonging to B1 and D phylogroups had predominantly at least one gene related to the autotransporter. Neither the gene coding capsule nor the toxin were included in A and B1 phylogroups.

### Genotyping with the PCR MP method

**Fig 4:**
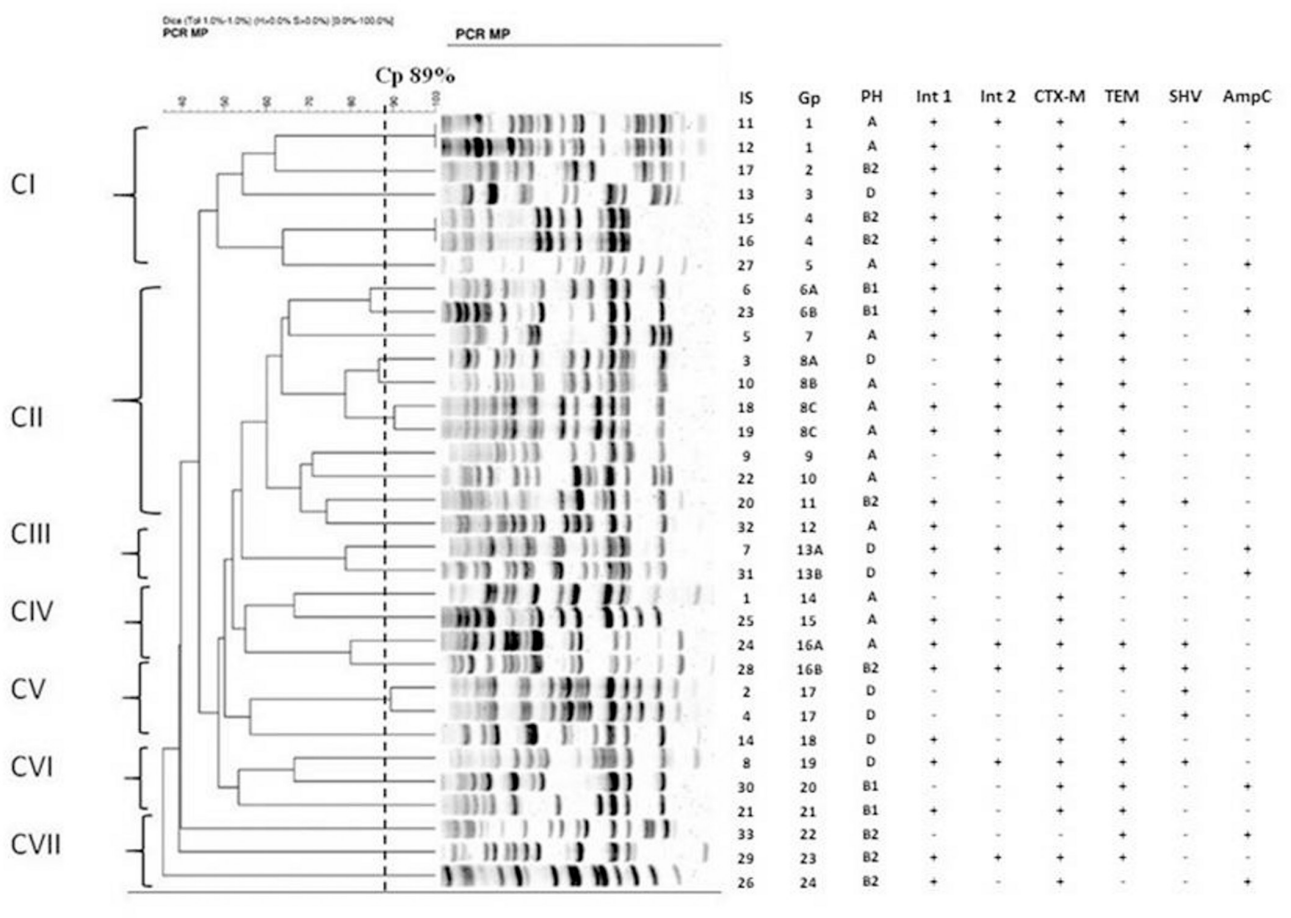
PCR MP band profiles for 33 *E*. *coli* strains isolated from GI-tract avian and phylogenetic relatedness. The number of isolates and name of PCR MP types are given for each lane. Isolates with Cp ≥89% were associated with the same genotype and for ≥80% subtypes. Legend: I-VII–clades; Gp–genotype; Is- number of isolate; PH- phylogenetic group; Int1, Int2 –class 1 and 2 integrases; CTX-M–*bla*_CTX-M_ gene; TEM- *bla*_TEM_−gene; SHV- *bla*_SHV_ gene; AMP- *bla*_AmpC_ gene.

The relationships between *E*. *coli* isolates were established by the PCR MP fingerprinting method (Fig 4). Phylogenetic analysis of 33 isolates (IS) showed seven clades (C) (I-VII) with 24 genotypes (Gp) and subtypes (designated A and B). Clade I (CI) contained 5 genotypes (marked Gp1-Gp5) for 7 isolates (Is11, Is12, Is13, Is15, Is16, Is17 and Is27). Isolates 11 and 12, with the Gp1 genotype were recovered from two wild Mallards captured in geographically separate locations at intervals of 11 weeks. Two morphologically dissimilar isolates, Is15 and Is16, from the cloacal swab of Eurasian Coot had the same Gp4 genotype.

The second clade (CII) with 11 isolates (Is3, Is5, Is6, Is9, Is10, Is18, Is19, Is20, Is22, Is23, Is32) had only seven genotypes designated as Gp6 with two subtypes Gp6A, Gp6B (isolates: Is6 from Common Gull and Is23 from Mallard), Gp7, Gp9-Gp12 (isolates: Is9, Is22, Is20, Is32), Gp8 (isolates: Is3 from Mallard, Is10 from Black-headed Gull, Is18 and Is19 from Eurasian Coot) with three subtypes Gp8A, Gp8B, Gp8C (2×), respectively. Interestingly, the unique number on the bird ring indicated that isolates Is18 and Is19, showing different colony morphology, were recovered from the same individual (Eurasian Coot) that had been caught four weeks earlier.

A group of two isolates (Is7 and Is31) considered together as a third clade (CIII) shared the same genotype consisting of the Gp13A and Gp13B subtypes. Both isolates, morphologically distinct, were collected from one bird (Common Gull).

The fourth clade (CIV) was assembled from 4 isolates, Is1, Is25, Is24 and Is28, with three genotypes: Gp14, Gp15 and Gp16, with A and B subtypes, respectively. Is24 and Is28 differed in colony morphology and size, and were obtained from a single swab collected from a Black-headed Gull. It is noteworthy that both isolates representing the same genotype belonged to different phylogroups, A and B2, respectively, and showed unique virulence and antimicrobial resistance profiles. Group B2 *E*. *coli* is not common among commensal intestinal flora and the presence within this group significantly more virulence factors is caused mainly by horizontal gene transfer (HGT).

Isolates Is2, Is4 and Is14, associated with genotypes Gp17 (2×) and Gp18, respectively, belong to the fifth clade (CV). Is2 and Is4 were recovered from two European Herring Gulls sampled at different locations at an interval of five weeks.

The sixth (CVI) and seventh (CVII) clades with three isolates each (Is8, Is21, Is30 and Is26, Is29, Is33, respectively) consist of the different genotypes marked from Gp19 to Gp24.

Strains carrying the same genotype are assigned to the same phylogroup and often share the same antibiotics resistance profile. However, some subtypes of the same genotype belong to different phylogroups and do not correspond to the same antibiotics resistance profile, which suggests HGT of the resistance genes.

## Discussion

Wild birds live in most habitats around the world and their faeces, containing potentially harmful bacteria, are freely dispersed in the environment. Bacteria causing urinary tract infections, sepsis and respiratory infections, as well as those associated with food poisoning, are common in the faeces of all birds [46]. A large recipient of antibiotic-resistant bacteria that come from different sources (e.g., bird droppings, medical waste, municipal wastewater, agricultural applications) is the aquatic environment. It also appears that birds roosting on surface water have an adverse effect on the microbiological quality of the water [47]. In connection with this, there is an increasing body of research demonstrating that the aquatic environment is an ideal setting for the acquisition and dissemination of antimicrobial resistance genes and virulence factors harboured by commensal and pathogenic bacteria [46].

Based on our research, we wanted to answer the following questions: 1. Are extended-spectrum β-lactamase*-*positive *Escherichia coli* common among wild birds living in Poland? 2. What VFs are carried by *E*. *coli* strains isolated from wild birds in Poland? 3. Which phylogenetic groups are typical for *E*. *coli* strains isolated from the GI-tract of wild birds? 4. Is there an association between phylogenetic groups with multidrug resistance and VFs in *E*. *coli* isolates from wild birds? 5. What is the genetic diversity of *E*. *coli* strains isolated from the GI-tract of wild birds living in the habitats of people?

### Wild birds as a reservoir of ESBL/AmpC *E*. *coli*

Among Enterobacterales, ESBL/AmpC-producing *E*. *coli* has been increasingly reported in Europe and other areas of the world. The CTX-M- type β-lactamases are the most common ESBLs among isolates of human and veterinary origin worldwide [48–51]. In our study a comparatively high proportion (13.7%) of wild birds were carriers of ESBL or/and AmpC-producing *E*. *coli* strains (S1 Table). This percentage correlates with results obtained from studies performed in Europe [52–55]. According to our report the highest share of ESBL strains was found in rallids and the lowest in corvids and ducks. This may reflect differences in diet and feeding among these groups. However, the rallids were captured in sites different from those where the other species with ESBL strains were caught. Hence, the region where the ringing of the birds takes place may be essential for the observed differences between bird families in ESBL/AmpC carriage. The most commonly identified resistance determinants were CTX-M and TEM. Characterization of the β-lactamase genes showed a dominance of the *bla*_CTX-M_ gene present in 87,9% of the ESBLs-producing *E*. *coli* isolates. Direct sequencing of amplicons, identified CTX-M-15 as the main CTX-M-type β-lactamase. A significant presence of CTX-M-producing species among strains isolated from wild birds has been reported in other studies [36, 54]. TEM- and SHV-type ESBLs were mostly prevalent among nosocomial *E*. *coli* isolates and not community-acquired pathogens [55–57]. The enormous spread of CTX-M-type ESBLs is rapidly changing ESBL epidemiology and, in many parts of the world, these enzymes are now the most prevalent ESBLs in Enterobacterales. In Europe, the CTX-M type has already taken the dominant position from TEM and SHV types [54, 55].

By identifying the same clones in humans and wild/domestic animals, it has been proven that the transmission of ESBL-producing strains between species is possible. The key factor for dissemination of ESBLs between bacteria, in both hospital and community settings, is HGT [48, 51]. A particular role in HGT play integrons. Many Enterobacterales that inhabit the intestinal tracts of humans and animals carry integrons as a consequence of the high consumption of antibiotics in human and veterinary medicine [58]. Class 1 integrons identified in a great number of bacterial genera appear to be prevalent. About 30% of all resistant *E*. *coli* recovered from cattle, pigs and poultry carried a class 1 integron [59]. The presence of integrons among resistant *E*. *coli* isolates from wild birds is rather low in contrast to hospital *E*. *coli* strains [60–63], however, in our study, 72% of the isolates of *E*. *coli* contained the class 1 integrase gene and as many as 51% of the isolates tested contained the class 2 integrase gene. Taking into consideration that wild birds do not naturally come into contact with antibiotics, it is remarkable that class 1 and 2 integrons were frequently detected among the isolates in this study.

### VFs are carried by *E*. *coli* strains isolated from wild birds in Poland

The results of this study showed that wild birds may be colonized by both commensal and pathogenic *E*. *coli* strains. To discriminate between pathogenic and commensal *E*. *coli* strains, the VFs *irp2*, *fyuA*, *kspMTII* and *fecA* that distinguished both pathotypes were studied. The *irp2*-*fyuA* gene cluster has been confirmed to be located on a high pathogenicity island (HPI) detected originally in *Yersinia* spp. [64]. Both genes are frequently found in pathogenic strains of *E*. *coli* [65–69]. Studies carried out in Brazil suggest that *irp2* could be a candidate for predicting the pathogenicity of avian *E*. *coli* strains, and *fyuA* may be related to virulence [70]. In our studies, genes *irp2* and *fyuA* were more often associated with pathogenic rather than commensal strains (81.3% and 29%, respectively), with differences statistically significant (P value 0.004 for both) (S2 Table).

On the basis of our results, we also infer that *kspMTII* may be a candidate for predicting the degree of *E*. *coli* strains pathogenicity. As reported, prevalence rates of the *kspMTII* gene for avian pathogenic *E*. *coli* isolates have tended to be low: 15.7% and 0% for American [71] and Zimbabwean isolates [72]. In our study the *kspMTII* gene was found more frequently among pathogenic group B2/D (37.5%) when compared with commensal strains from group A/B1 (0%), with differences statistically significant (P value 0.007).

### The phylogenetic groups of *E*. *coli* strains isolated from the GI-tract of wild birds

Based on the genetic structure of the *E*. *coli* population in animals and humans, the association between the phylogroup strain and host and environmental factors has been demonstrated [73–75]. Most of avian *E*. *coli* strains are assigned to phylogroups A, B1 while the majority of the extraintestinal pathogenic *E*. *coli* isolates of human origin are associated with phylogenetic type B2 and to a lesser degree to group D [24, 25]. Diet, gut morphology and body mass appear to be crucial predictors of the distribution of the phylogenetic groups [76]. In animals, the main factor influencing the genetic structure of the *E*. *coli* population in the intestine is domestication. Domestic animals have a lower percentage of B2 strains than their wild counterparts (from 30% in wild animals to 14% and 11% in livestock and zoo animals, respectively) and a higher percentage of strains belonging to the phylogenetic group A [77–79]. In our research, we conclude that the wild birds were colonized both by non-pathogenic strains (with the advantage of A group) and by pathogenic strains belonging to groups B2 and D (24,24% each). The most commonly detected pathogenic human ExPEC strains belong to group B2, which suggests that avian *E*. *coli* had a hospital origin.

The strains isolated from gulls (Laridae) were mostly classified as D or B2 phylogroups, similarly, the strains isolated from Rallidae predominantly belong to the B2 phylogroup (62,5%). Gulls are mainly omnivorous and in the non-breeding season they are associated with human settlements where they often feed on litter of human origin [76, 80] and may have been in contact with human ExPEC strains [37]. Rallidae, on the other hand, remain only on bodies of water and have limited contact with anthropogenic waste. It seems that the presence of B2 ExPEC strains is not related to environmental selectivity or the feeding ecology of these bird families, but rather these strains are widespread in the urban environment as they were found in the samples collected in different urban areas.

### An association between phylogenetic groups with multidrug resistance and virulence in *E*. *coli* isolates from wild birds

Our research showed that most of the ESBL genes were found in the phylogenetic group B2, despite the fact that this group was not the most prevalent. Recent reports suggest that the *E*. *coli* strains belonging to the B2 phylogroup produce multiple VFs, which influences their pathogenic potential [16, 56, 81]. In our research 6 VFs (*vat*, *tosA*, *tosB*, *hly*, *usp* and *sfa*) out of 28 VFs have only been detected in phylogroup B2. *tosA* and *tosB* genes are considered as markers for the presence of other virulence genes, whereas, *usp*, *hlyA*, *sfa* genes are often detected in the UPEC strains responsible for UTI [16, 82]. Taking into account the division into commensal and pathogenic strains it was observed that the *fecA* gene encoding the outer membrane receptor with siderophore uptake transmembrane transporter activity was significantly associated with the commensal group of isolates (A, B1). Three other genes—*kspMTII*, *irp2* and *fyuA* were mainly associated with the pathogenic group of isolates (B2, D). *irp2* and *fyuA* genes are also found in avian pathogenic *Escherichia coli* (APEC). A third gene–*kspMTII*, belongs to a group of genes responsible for the synthesis of capsules. Gene coding capsules are most frequently found in isolates from community environments compared to hospital samples [83]. The relationship between resistance and virulence traits is mainly observed among clinical strains in the hospital environment [81, 84, 85]. In our research, an intriguing phenomenon is that *E*. *coli* strains producing ESBL/AmpC showing a high virulence profile, were also present in the natural habitat of birds. The dissemination of these strains via birds may also suggest an increased contamination of the human environment.

### Assessment of genetic diversity of *E*. *coli* strains isolated from the GI-tract by genotyping

The PCR MP technique is widely used in various aspects of epidemiological study due to its high discriminatory power [56, 86–88]. We decided to use this method for studying the diversity of ESBL/AmpC strains in the environment. We demonstrated the genetic heterogeneity and diverse origins of *E*. *coli* strains (24 genotypes with subtypes). ESBL/AmpC positive strains of the same genotype successfully colonized the birds of different species. Migrating birds can transfer them to other areas (and in turn to birds), so they are a transmission vector of ESBL strains between different water bodies. In our study two *E*. *coli* isolates collected from a Eurasian Coot, demonstrating phenotypic diversity in colony morphology and size, had the same genotypic profile. Interestingly, six weeks later, the same individual was caught again in the same area and once more two *E*. *coli* colonies, with distinct coloration and morphology, exhibiting an identical PCR-MP profile, were recovered. In both cases isolates were confirmed as positive for ESBL production but with a different genotypic profile. Repeated findings of ESBL/AmpC-positive isolates over time indicate that these strains are resident and circulate abundantly in the environment. We also found that two *E*. *coli* isolates recovered from two Mallards captured in different locations exhibited an identical PCR MP profile. This identification of a common clone at two geographically distant centres indicated the possibility of transmission of ESBL/AmpC producing *E*. *coli* in the absence of antibiotic selection. The residence time of resistant bacteria in the digestive tract of wild birds remains unclear.

### Conclusion

Free-living animals may serve as a reservoir and source of antibiotic-resistant bacteria, however, they have been studied much less frequently than farm animals. Our work revealed that a broad range of avian species were identified as highly prevalent carriers of multi-resistant *E*. *coli*. Our research indicates that not only the B2/D phylogenetic group but also the commensal A1 group is equipped with virulence genes and fitness factors. The presence of VFs along with the cephalosporin resistance AmpC gene and genes encoding ESBL may indicate the appearance of so-called high-risk strains in the animal environment. The spread between bacteria of antibiotic resistance genes is associated with the presence of mobile genetic elements that enable HGT among bacteria. It leads to the genetic variation of microorganisms, playing a major role in the evolution of bacteria. Understanding the scale of occurrence of drug-resistance strains in the environment, and in particular among birds, will allow for the assessment of the transmission of hospital strains into the environment and provide an indication of the risk of acquiring these strains by people from particularly exposed groups. Depending on resistance profiles and the prevalence of virulence factors, the *E*. *coli* strains of our study could have a considerable potential to cause human disease.

## Supporting information

S1 Table Prevalence (%) of avian ESBL/AmpC *E*. *coli* strains in different bird families. (DOCX)

S2 Table Prevalence of virulence genes among *E*. *coli* isolates related to the commensal (A, B1) and pathogenic (B2, D) phylogroups. (DOCX)

## References

[1] Woodford N, Turton JF, Livermore DM. Multiresistant Gram-negative bacteria: The role of high-risk clones in the dissemination of antibiotic resistance. FEMS Microbiology Rev. 2011;35:736–55. doi: 10.1111/j.1574-6976.2011.00268.x 21303394

[2] Martínez-Martínez L, González-López JJ. Carbapenemases in *Enterobacteriaceae*: types and molecular epidemiology. Enferm Infecc Microbiol Clin. 2014;32:4–9. doi: 10.1016/S0213-005X(14)70168-5 25542046

[3] Cantón R, González-Alba JM, Galán JC. CTX-M enzymes: origin and diffusion. Front. Microbiol. 2012;3:110. doi: 10.3389/fmicb.2012.00110 22485109 PMC3316993

[4] Handrova L, Kmet V. Antibiotic resistance and virulence factors of *Escherichia coli* from eagles and goshawks. Pesticides, Food Contaminants, and Agricultural Wastes. 2019;54:605–614. doi: 10.1080/03601234.2019.1608103 31046564

[5] Nicolas-Chanoine MH, Bertrand X, Madec JY. *Escherichia coli* st131, an intriguing clonal group. Clin Microbiol Rev. 2014;27:534–74. doi: 10.1128/CMR.00125-13 24982321 PMC4135899

[6] Zhu Ge X, Jiang J, Pan Z, Hu L, Wang S, Wang H, et al. Comparative genomic analysis shows that avian pathogenic *Escherichia coli* isolate IMT5155 (O2:K1:H5; ST Complex 95, ST140) shares close relationship with ST95 APEC O1:K1 and human ExPEC O18:K1 strains. PLoS One. 2014;9:e112048. doi: 10.1371/journal.pone.0112048 25397580 PMC4232414

[7] Džidić S, Šušković J, Kos B. Antibiotic resistance mechanisms in bacteria: biochemical and genetic aspects. Food Technol Biotechnol. 2008;46:11–21.

[8] Moulin-Schouleur M, Répérant M, Laurent S, Brée A, Mignon-Grasteau S, Germon P, et al. Extraintestinal pathogenic *Escherichia coli* strains of avian and human origin: Link between phylogenetic relationships and common virulence patterns. J Clin Microbiol. 2007;45:3366–3376. doi: 10.1128/JCM.00037-07 17652485 PMC2045314

[9] Vounba P, Arsenault J, Bada-Alambédji R, Fairbrother JM. Prevalence of antimicrobial resistance and potential pathogenicity, and possible spread of third generation cephalosporin resistance, in *Escherichia coli* isolated from healthy chicken farms in the region of Dakar, Senegal. PLOS ONE 2019;14:e0214304. doi: 10.1371/journal.pone.0214304 30913237 PMC6435184

[10] Sarowska J, Futoma-Koloch B, Jama-Kmiecik A, Frej-Madrzak M, Ksiazczyk M, Bugla-Ploskonska G, et al. Virulence factors, prevalence and potential transmission of extraintestinal pathogenic *Escherichia coli* isolated from different sources: recent reports. Gut Pathogens. 2019;11:10 doi: 10.1186/s13099-019-0290-0 30828388 PMC6383261

[11] Searle LJ, Méric G, Porcelli I, Sheppard SK, Lucchini S. Variation in siderophore biosynthetic gene distribution and production across environmental and faecal populations of *Escherichia coli*. PLoS One. 2015;10:e0117906. doi: 10.1371/journal.pone.0117906 25756870 PMC4355413

[12] Gao Q, Wang X, Xu H, Xu Y, Ling J, Zhang D, et al. Roles of iron acquisition systems in virulence of extraintestinal pathogenic *Escherichia coli*: Salmochelin and aerobactin contribute more to virulence than heme in a chicken infection model. BMC Microbiol. 2012;12:143. doi: 10.1186/1471-2180-12-143 22817680 PMC3496646

[13] Ewers C, Antão EM, Diehl I, Philipp HC, Wieler LH. Intestine and environment of the chicken as reservoirs for extraintestinal pathogenic *Escherichia coli* strains with zoonotic potential. Appl Environ Microbiol. 2009;75:184–192. doi: 10.1128/AEM.01324-08 18997030 PMC2612213

[14] Mitchell NM, Johnson JR, Johnston B, Curtiss R, Mellata M. Zoonotic potential of *Escherichia coli* isolates from retail chicken meat products and eggs. Appl Environ Microbiol. 2015;81:1177–1187. doi: 10.1128/AEM.03524-14 25480753 PMC4292506

[15] Clermont O, Christenson JK, Denamur E, Gordon DM. The Clermont *Escherichia coli* phylo-typing method revisited: improvement of specificity and detection of new phylo-groups. Environ Microbiol Rep. 2013;5:58–65. doi: 10.1111/1758-2229.12019 23757131

[16] Krawczyk B, Sledzinska A, Szemiako K, Samet A, Nowicki B, Kur J. Characterisation of *Escherichia coli* isolates from the blood of haematological adult patients with bacteraemia: translocation from gut to blood requires the cooperation of multiple virulence factors. Eur J Clin Microbiol Infect Dis. 2015;34:1135–1143. doi: 10.1007/s10096-015-2331-z 25655758 PMC4426128

[17] Krawczyk B, Sledzinska A, Piekarska A, Hellmann A, Kur J. Recurrent bowel-blood translocations of *Escherichia coli* with the unique virulence characteristics over three-year period in the patient with acute myeloid leukaemia–case report. J Appl Genet. 2017;58:415–418. doi: 10.1007/s13353-017-0393-6 28324282 PMC5509818

[18] Michalik M, Samet A, Marszalek A, Krawczyk B, Kotlowski R, Nowicki A, et al. Intra-operative biopsy in chronic sinusitis detects pathogenic *Escherichia coli* that carry *fim*G/H, *fyu*A and *agn*43 genes coding biofilm formation. PLoS One. 2018;13: e0192899. doi: 10.1371/journal.pone.0192899 29570706 PMC5865710

[19] Yair Y, Gophna U. Pandemic bacteremic *Escherichia coli* strains: evolution and emergence of drug-resistant pathogens. Curr Top Microbiol Immunol. 2018;416:163–180. doi: 10.1007/82_2018_109 30046983

[20] Atterby C, Börjesson S, Ny S, Järhult JD, Byfors S, Bonnedahl J. ESBL-producing *Escherichia coli* in Swedish gulls—A case of environmental pollution from humans? PLoS One. 2017;12:e0190380. doi: 10.1371/journal.pone.0190380 29284053 PMC5746268

[21] Grönthal T, Österblad M, Eklund M, Jalava J, Nykäsenoja S, Pekkanen K, et al. Sharing more than friendship–transmission of NDM-5 ST167 and CTX-M-9 ST69 *Escherichia coli* between dogs and humans in a family, Finland, 2015. Euro Surveill. 2018;23:pii = 1700497. doi: 10.2807/1560-7917.ES.2018.23.27.1700497 PMC6152158 29991384

[22] Radhouani H, Silva N, Poeta P, Torres C, Correia S, Igrejas G. Potential impact of antimicrobial resistance in wildlife, environment, and human health. Front. Microbiol. 2014;5:23. doi: 10.3389/fmicb.2014.00023 24550896 PMC3913889

[23] Röderova M, Halova D, Papousek I, Dolejska M, Masarikova M, Hanulik V, et al. Characteristics of quinolone resistance in *Escherichia coli* isolates from humans, animals, and the environment in the Czech Republic. Front Microbiol. 2017;7:2147. doi: 10.3389/fmicb.2016.02147 28119674 PMC5220107

[24] Köhler CD, Dobrindt U. What defines extraintestinal pathogenic *Escherichia coli*? J Med Microbiol. 2011;301:642–647. doi: 10.1016/j.ijmm.2011.09.006 21982038

[25] Tivendale KA, Logue CM, Kariyawasam S, Jordan D, Hussein A, Li G, et al. Avian-pathogenic *Escherichia coli* strains are similar to neonatal meningitis *E*. *coli* strains and are able to cause meningitis in the rat model of human disease. Infect Immun. 2010;78:3412–3419. doi: 10.1128/IAI.00347-10 20515929 PMC2916289

[26] Furness R. Seabirds as monitors of the marine environment. ICES J Mar Sci. 1997;54:726–737. doi: 10.1006/jmsc.1997.0243

[27] Binkowski LJ, Meissner W. Levels of metals in blood samples from Mallards (*Anas platyrhynchos*) from urban areas in Poland. Environ Pollut. 2013;178:336–342. doi: 10.1016/j.envpol.2013.03.030 23603471

[28] Dynowska M, Meissner W, Pacynska J. Mallard duck (*Anas platyrhyncho*s) as a potential link in the epidemiological chain mycoses originating from water reservoirs. Bull Vet Inst Pulawy. 2013;57:323–328. doi: 10.2478/bvip-2013-0056

[29] Smits JEG, Fernie KJ. Avian wildlife as sentinels of ecosystem health. Comp Immunol Microbiol Infect Dis. 2013;36:333–342. doi: 10.1016/j.cimid.2012.11.007 23260372

[30] Tsiodras S, Kelesidis T, Kelesidis I, Bauchinger U, Falagas ME. Human infections associated with wild birds. J Infect. 2008;56:83–98. doi: 10.1016/j.jinf.2007.11.001 18096237 PMC7172416

[31] Bonnedahl J, Järhult JD. Antibiotic resistance in wild birds. Ups J Med Sci. 2014;119:113–116. doi: 10.3109/03009734.2014.905663 24697355 PMC4034547

[32] Dolejská M, Bierosová B, Kohoutová L, Literák I, Cízek A. Antibiotic-resistant *Salmonella* and *Escherichia coli* isolates with integrons and extended-spectrum beta-lactamases in surface water and sympatric black-headed gulls. J Appl Microbiol. 2009;106:1941–50. doi: 10.1111/j.1365-2672.2009.04155.x 19245407

[33] Shobrak MY, Abo-Amer AE. Role of wild birds as carriers of multi-drug resistant *Escherichia coli* and *Escherichia vulneris*. Braz J Microbiol. 2014;45:1199–1209. doi: 10.1590/s1517-83822014000400010 25763023 PMC4323292

[34] European Committee on Antimicrobial Susceptibility Testing. Breakpoints tables for interpretation of MICs and zones diameters. Version 10.0, 2020. 01-01-2020. Available from: http://www.eucast.org.

[35] Birnboim HC, Doly J. A rapid alkaline extraction procedure for screening recombinant plasmid DNA. Nucleic Acids Res. 1979;7:1513–1523. doi: 10.1093/nar/7.6.1513 388356 PMC342324

[36] Literak I, Dolejska M, Janoszowska D, Hrusakova J, Meissner W, Rzyska H, et al. Antibiotic-resistant escherichia coli bacteria, including strains with genes encoding the extended-spectrum beta-lactamase and QnrS, in waterbirds on the baltic sea coast of Poland. Appl Environ Microbiol. 2010;76:8126–8134. doi: 10.1128/AEM.01446-10 20952638 PMC3008254

[37] Literak I, Micudova M, Tausova D, Cizek A, Dolejska M, Papousek I, et al. Plasmid-mediated quinolone resistance genes in fecal bacteria from rooks commonly wintering throughout Europe. Microb Drug Resist. 2012;18:567–573. doi: 10.1089/mdr.2012.0075 22731858

[38] Krawczyk B, Samet A, Leibner J, Sledzinska A, Kur J. Evaluation of a PCR melting profile technique for bacterial strain differentiation. J Clin Microbiol. 2006;44:2327–2332. doi: 10.1128/JCM.00052-06 16825344 PMC1489531

[39] Edelstein M, Pimkin M, Palagin I, Edelstein I, Stratchounski L. Prevalence and molecular epidemiology of CTX-M extended-spectrum β-lactamase-producing *Escherichia coli* and *Klebsiella pneumoniae* in Russian hospitals. Antimicrob Agents Chemother. 2003;47:3724–3732. doi: 10.1128/AAC.47.12.3724-3732.2003 14638473 PMC296190

[40] Krawczyk B, Michalik M, Fordon M, Wysocka M, Samet A, Nowicki B. *Escherichia coli* strains with virulent factors typical for uropathogens were isolated from sinuses from patients with chronic rhinosinusitis—case report. Pathogens. 2020;9:318. doi: 10.3390/pathogens9050318 32344929 PMC7280992

[41] Kotlowski R, Bernstein CN, Sepehri S, Krause DO. High prevalence of *Escherichia coli* belonging to the B2+D phylogenetic group in inflammatory bowel disease. Gut. 2007;56:669–675. doi: 10.1136/gut.2006.099796 17028128 PMC1942160

[42] Goldstein A, Kapelner A, Bleich J, Pitkin E. Peeking inside the black box: visualizing statistical learning with plots of individual conditional expectation. J Comput Graph Stat. 2015;24:44–65.

[43] Wickham H. ggplot2: Elegant graphics for data analysis. In New York: Springer-Verlag; 2009.

[44] de Vries A. ggdendro: Create dendrograms and tree diagrams using ’ggplot2’. 2020. https://cran.r-project.org/web/packages/ggdendro/ggdendro.pdf.

[45] Sievert DM, Ricks P, Edwards JR, Schneider A, Patel J, Srinivasan A, et al. National Healthcare Safety Network (NHSN) Team and Participating NHSN Facilities. Antimicrobial-resistant pathogens associated with healthcare-associated infections summary of data reported to the National Healthcare Safety Network at the Centers for Disease Control and Prevention, 2009–2010. Infect Control Hosp Epidemiol. 2013;34:1–14. doi: 10.1086/668770 23221186

[46] Huerta B, Marti E, Gros M, López P, Pompêo M, Armengol J, et al. Exploring the links between antibiotic occurrence, antibiotic resistance, and bacterial communities in water supply reservoirs. Sci Total Environ. 2013;456:161–170. doi: 10.1016/j.scitotenv.2013.03.071 23591067

[47] Smolders A, Smolders K, Watkinson A, Ryder D. Reassessing the risk of microbial contamination from roosting cormorants in source water supply reservoirs. Lake and Reservoir Management. 2014;30:23–31. doi: 10.1080/10402381.2013.866997

[48] Pietsch M, Eller C, Wendt C, Holfelder M, Falgenhauer L, Fruth A, et al. Molecular characterisation of extended-spectrum β-lactamase (ESBL)-producing *Escherichia coli* isolates from hospital and ambulatory patients in Germany. Vet Microbiol. 2017;200:130–137. doi: 10.1016/j.vetmic.2015.11.028 26654217

[49] Wang S, Zhao SY, Xiao SZ, Gu FF, Liu QZ, Tang J, et al. Antimicrobial resistance and molecular epidemiology of *Escherichia coli* causing bloodstream infections in three hospitals in Shanghai, China. PLoS One. 2016;11:e0147740. doi: 10.1371/journal.pone.0147740 26824702 PMC4733056

[50] Wellington EM, Boxall AB, Cross P, Feil EJ, Gaze WH, Hawkey PM, et al. The role of the natural environment in the emergence of antibiotic resistance in Gram-negative bacteria. Lancet Infect Dis. 2013;13:155–165. doi: 10.1016/S1473-3099(12)70317-1 23347633

[51] Liu BT, Yang QE, Li L, Sun J, Liao XP, Fang LX, et al. Dissemination and characterization of plasmids carrying oqxAB-blaCTX-M genes in *Escherichia coli* isolates from food-producing animals. PLoS One. 2013;8:e73947. doi: 10.1371/journal.pone.0073947 24040123 PMC3767592

[52] Gordon DM, Clermont O, Tolley H, Denamur E. Assigning *Escherichia coli* strains to phylogenetic groups: multi-locus sequence typing versus the PCR triplex method. Environ Microbiol. 2008;10:2484–2496. doi: 10.1111/j.1462-2920.2008.01669.x 18518895

[53] Alcalá L, Alonso CA, Simón C, González-Esteban C, Orós J, Rezusta A, et al. Wild Birds, Frequent carriers of extended-spectrum β-lactamase (ESBL) producing *Escherichia coli* of CTX-M and SHV-12 yypes. Microb Ecol. 2016;72:861–869. doi: 10.1007/s00248-015-0718-0 26687342

[54] Stedt J, Bonnedahl J, Hernandez J, Waldenström J, McMahon BJ, Tolf C, et al. Carriage of CTX-M type extended spectrum β-lactamases (ESBLs) in gulls across Europe. Acta Vet Scand. 2015;57:74. doi: 10.1186/s13028-015-0166-3 26526188 PMC4629291

[55] Baraniak A, Fiett J, Sulikowska A, Hryniewicz W, Gniadkowski M. Countrywide spread of CTX-M-3 extended-spectrum β-lactamase-producing microorganisms of the family *Enterobacteriaceae* in Poland. Antimicrob Agents Chemother. 2002;46:151–159. doi: 10.1128/AAC.46.1.151-159.2002 11751126 PMC126981

[56] Izdebski R, Baraniak A, Fiett J, Adler A, Kazma M, Salomon J, et al. Clonal structure, extended-spectrum β-lactamases,and acquired AmpC-type cephalosporinases of *Escherichia coli* populations colonizing patients in rehabilitation centers in four countries. Antimicrob Agents Chemother. 2013;57:309–316. doi: 10.1128/AAC.01656-12 23114774 PMC3535924

[57] Livermore DM, Canton R, Gniadkowski M, Nordmann P, Rossolini GM, Arlet G, et al. CTX-M: Changing the face of ESBLs in Europe. J Antimicrob Chemother. 2007;59:165–174. doi: 10.1093/jac/dkl483 17158117

[58] Carattoli A, Tosini F, Giles WP, Rupp ME, Hinrichs SH, Angulo FJ, et al. Characterization of plasmids carrying CMY-2 from expanded-spectrum cephalosporin-resistant *Salmonella* strains isolated in the United States between 1996 and 1998. Antimicrob Agents Chemother. 2002;46:1269–1272. doi: 10.1128/AAC.46.5.1269-1272.2002 11959555 PMC127137

[59] Guerra B, Junker E, Schroeter A, Malorny B, Lehmann S, Helmuth R. Phenotypic and genotypic characterization of antimicrobial resistance in German *Escherichia coli* isolates from cattle, swine and poultry. J Antimicrob Chemother. 2003;52:489–92. doi: 10.1093/jac/dkg362 12888584

[60] Infante B, Grape M, Larsson M, Kristiansson C, Pallecchi L, Rossolini GM, et al. Acquired sulphonamide resistance genes in faecal *Escherichia coli* from healthy children in Bolivia and Peru. Int J Antimicrob Agents. 2005;25:308–312. doi: 10.1016/j.ijantimicag.2004.12.004 15784310

[61] Leverstein-van Hall MA, Paauw A, Box ATA, Blok HEM, Verhoef J, Fluit AC. Presence of integron-associated resistance in the community is widespread and contributes to multidrug resistance in the hospital. J Clin Microbiol. 2002;40:3038–40. doi: 10.1128/JCM.40.8.3038-3040.2002 12149373 PMC120645

[62] Machado E, Cantón R, Baquero F, Galán JC, Rollán A, Peixe L, et al. Integron content of extended-spectrum-β-lactamase-producing *Escherichia coli* strains over 12 years in a single hospital in Madrid, Spain. Antimicrob Agents Chemother. 2005;49:1823–1829. doi: 10.1128/AAC.49.5.1823-1829.2005 15855502 PMC1087637

[63] Skurnik D, Le Menac’h A, Zurakowski D, Mazel D, Courvalin P, Denamur E, et al. Integron-associated antibiotic resistance and phylogenetic grouping of *Echerichia coli* isolates from healthy subjects free of recent antibiotic exposure. Antimicrob Agents Chemother. 2005;49:3062–3065. doi: 10.1128/AAC.49.7.3062-3065.2005 15980401 PMC1168629

[64] Carniel E, Guilvout I, Prentice M. Characterization of a large chromosomal “high-pathogenicity island” in biotype 1B *Yersinia enterocolitica*. J Bacteriol. 1996;178:6743–6751. doi: 10.1128/jb.178.23.6743-6751.1996 8955291 PMC178570

[65] Ewers C, Janßen T, Kießling S, Philipp HC, Wieler LH. Molecular epidemiology of avian pathogenic *Escherichia coli* (APEC) isolated from colisepticemia in poultry. Vet Microbiol. 2004;104:91–101. doi: 10.1016/j.vetmic.2004.09.008 15530743

[66] Gophna U, Oelschlaeger TA, Hacker J, Ron EZ. *Yersinia* HPI in septicemic *Escherichia coli* strains isolated from diverse hosts. FEMS Microbiol Lett. 2001;196:57–60. doi: 10.1111/j.1574-6968.2001.tb10540.x 11257548

[67] Johnson JR, Clabots C, Kuskowski MA. Multiple-host sharing, long-term persistence, and virulence of *Escherichia coli* clones from human and animal household members. J Clin Microbiol. 2008;46:4078–4082. doi: 10.1128/JCM.00980-08 18945846 PMC2593269

[68] Johnson JR, Delavari P, Kuskowski M, Stell AL. Phylogenetic distribution of extraintestinal virulence-associated traits in *Escherichia coli*. J Infect Dis. 2001;183:78–88. doi: 10.1086/317656 11106538

[69] Schubert S, Rakin A, Karch H, Carniel E, Heesemann J. Prevalence of the “high-pathogenicity island” of *Yersinia* species among *Escherichia coli* strains that are pathogenic to humans. Infect Immun. 1998;66:480–485. doi: 10.1128/IAI.66.2.480-485.1998 9453599 PMC107931

[70] Jørgensen SL, Stegger M, Kudirkiene E, Lilje B, Poulsen LL, Ronco T, et al.Diversity and population overlap between avian and human *Escherichia coli* belonging to sequence type 95. mSphere. 2019;4: e00333–18. doi: 10.1128/mSphere.00333-18 30651401 PMC6336079

[71] Johnson TJ, Wannemuehler Y, Johnson SJ, Stell AL, Doetkott C, Johnson JR, et al. Comparison of extraintestinal pathogenic *Escherichia coli* strains from human and avian sources reveals a mixed subset representing potential zoonotic pathogens. Appl Environ Microbiol. 2008;74:7043–7050. doi: 10.1128/AEM.01395-08 18820066 PMC2583479

[72] van der Westhuizen WA, Bragg RR. Multiplex polymerase chain reaction for screening avian pathogenic *Escherichia coli* for virulence genes. Avian Pathol. 2012;41:33–40. doi: 10.1080/03079457.2011.631982 22845319

[73] Gordon DM, Cowling A. The distribution and genetic structure of *Escherichia coli* in Australian vertebrates: Host and geographic effects. Microbiology. 2003;149:3575–3586. doi: 10.1099/mic.0.26486-0 14663089

[74] Savageau MA. *Escherichia coli* habitats, cell types, and molecular mechanisms of gene control. Am Nat. 1983;122:732–744. doi: 10.1086/284168

[75] Solo-Gabriele HM, Wolfert MA, Desmarais TR, Palmer CJ. Sources of *Escherichia coli* in a coastal subtropical environment. Appl Environ Microbiol. 2000;66:230–7. doi: 10.1128/AEM.66.1.230-237.2000 10618229 PMC91811

[76] Ewins PJ, Weseloh D V, Groom JH, Dobos RZ, Mineau P. The diet of Herring Gulls (*Larus argentatus*) during winter and early spring on the lower Great Lakes. Hydrobiologia. 1994;279–280:39–55. doi: 10.1007/BF00027839

[77] Smith JL, Fratamico PM, Gunther NW. Extraintestinal pathogenic *Escherichia coli*. Foodborne Pathog Dis. 2007;4:134–63. doi: 10.1089/fpd.2007.0087 17600482

[78] Dobiasova H, Dolejska M, Jamborova I, Brhelova E, Blazkova L, Papousek I, et al. Extended spectrum beta-lactamase and fluoroquinolone resistance genes and plasmids among *Escherichia coli* isolates from zoo animals, Czech Republic. FEMS Microbiol Ecol. 2013;85:604–611. doi: 10.1111/1574-6941.12149 23679004

[79] Guenther S, Ewers C, Wieler LH. Extended-spectrum beta-lactamases producing *E*. *coli* in wildlife, yet another form of environmental pollution? Front Microbiol. 2011;2:246. doi: 10.3389/fmicb.2011.00246 22203818 PMC3244693

[80] Washburn BE, Bernhardt GE, Kutschbach-Brohl L, Chipman RB, Francoeur LC. Foraging Ecology of Four Gull Species at a Coastal–Urban Interface. Condor. 2013;115:67–76. doi: 10.1525/cond.2013.110185

[81] Jacoby GA. AmpC b-lactamases. Clin Microbiol Rev. 2009;22:161–182. doi: 10.1128/CMR.00036-08 19136439 PMC2620637

[82] Ong CLY, Ulett GC, Mabbett AN, Beatson SA, Webb RI, Monaghan W, et al. Identification of type 3 fimbriae in uropathogenic *Escherichia coli* reveals a role in biofilm formation. J Bacteriol. 2008;190:1054–63. doi: 10.1128/JB.01523-07 18055599 PMC2223576

[83] Souza GM, Neto, da Silva AM, Iacia MVM, Rodrigues MVP, Pereira VC, et al. Comparative study of genetic diversity, virulence genotype, biofilm formation and antimicrobial resistance of uropathogenic *Escherichia coli* (UPEC) isolated from nosocomial and community acquired urinary tract infections. Infect Drug Resist. 2019;12:3595–3606. doi: 10.2147/IDR.S228612 31819543 PMC6878931

[84] Briongos-Figuero LS, Gómez-Traveso T, Bachiller-Luque P, Domínguez-Gil González M, Gómez-Nieto A, Palacios-Martín T, et al. Epidemiology, risk factors and comorbidity for urinary tract infections caused by extended-spectrum beta-lactamase (ESBL)-producing enterobacteria. Int J Clin Pract. 2012;66:891–6. doi: 10.1111/j.1742-1241.2012.02991.x 22897466

[85] Picozzi S, Ricci C, Gaeta M, MacChi A, Dinang E, Paola G, et al. Do we really know the prevalence of multi-drug resistant *Escherichia coli* in the territorial and nosocomial population? Urol Ann. 2013;5:25–29. doi: 10.4103/0974-7796.106962 23662006 PMC3643319

[86] Krawczyk B, Leibner J, Baranska-Rybak W, Samet A, Nowicki R, Kur J. ADSRRS-fingerprinting and PCR MP techniques for studies of intraspecies genetic relatedness in *Staphylococcus aureus*. J Microbiol Methods. 2007;71:114–22. doi: 10.1016/j.mimet.2007.08.010 17889385

[87] Stojowska K, Kaluzewski S, Krawczyk B. Usefulness of PCR melting profile method for genotyping analysis of *Klebsiella oxytoca* isolates from patients of a single hospital unit. Polish J Microbiol. 2009;58:247–53. 19899618

[88] Sledzinska A, Samet A, Bronk M, Rybak B, Kur J, Krawczyk B, et al. *Escherichia coli* a forgotten pathogen in septicemia. Przegl Epidemiol. 2006;60:27–34. 16758735

