# Supplementary material for: Antibiotic resistance, virulence, and phylogenetic analysis of Escherichia coli strains isolated from free-living birds in human habitats
Source: PLoS One. 2022 Jan 12;17(1):e0262236. doi: 10.1371/journal.pone.0262236 (PMC8754294; doi:10.1371/journal.pone.0262236)
Supplement: S1 Table — (DOCX) [file pone.0262236.s001.docx]

Supporting Information

**S1 Table.** **Prevalence (%) of avian ESBL/AmpC *E. coli* strains in different bird families.**

| Family | Species | Number  of individuals  analyzed | Number of birds with ESBL/AmpC *E*. *coli*  (type of β- lactamase) |
| --- | --- | --- | --- |
| Anatidae | Mallard (*Anas platyrhynchos*) | 128 | 11 |
|  | Mute swan (*Cygnus olor*) | 5 | - |
|  | Mandarin duck (*Aix galericulata*) | 1 | - |
| Laridae | Black-headed gull (*Chroicocephalus ridibundus*) | 38 | 4 |
|  | European herring gull (*Larus argentatus*) | 16 | 3 |
|  | Common gull (*Larus canus*) | 12 | 4 |
| Corvidae | Hooded crow (*Corvus cornix*) | 5 | - |
|  | Jackdaw (*Corvus monedula*) | 4 | 1 |
|  | Rook (*Corvus frugilegus*) | 2 | - |
| Rallidae | Eurasian coot (*Fulica atra*) | 29 | 10 |
|  | Common moorhen (*Gallinula chloropus*) | 1 | - |
| Total | | 241 | 33 |
