# Supplementary material for: Antibiotic resistance, virulence, and phylogenetic analysis of Escherichia coli strains isolated from free-living birds in human habitats
Source: PLoS One. 2022 Jan 12;17(1):e0262236. doi: 10.1371/journal.pone.0262236 (PMC8754294; doi:10.1371/journal.pone.0262236)
Supplement: S2 Table — (DOCX) [file pone.0262236.s002.docx]

Supporting Information

**S2 Table**. **Prevalence of virulence genes among *E. coli* isolates related to the commensal (A, B1) and pathogenic (B2, D) phylogroups.**

| Category | Gene^1^ | Gene prevalence (% of isolates) | | | *P* value^2^ (Commensal vs. Pathogenic) |
| --- | --- | --- | --- | --- | --- |
|  |  | All isolates (n=33) | Commensal isolates (A, B1) (n=17) | Pathogenic isolates (B2, D) (n=16) |  |
| Adhesion | *fimH* | 23 (69.7) | 12 (70.6) | 11 (68.8) | 0.603 |
|  | *mrkD* | 4 (12) | 3 (17.7) | 1 (6.3) | 0.324 |
|  | *sfa* | 1 (3) | 0 (0) | 1 (6.3) | 0.485 |
|  | *papG* | 2 (6) | 1 (5.9) | 1 (6.3) | 0.742 |
|  | *afa/Dr* | 0 (0) | 0 (0) | 0 (0) | 1.0 |
|  | *ibeA* | 0 (0) | 0 (0) | 0 (0) | 1.0 |
|  | *focG* | 0 (0) | 0 (0) | 0 (0) | 1.0 |
| Toxins | *usp* | 1 (3) | 0 (0) | 1 (6.3) | 0.485 |
|  | *hly* | 1 (3) | 0 (0) | 1 (6.3) | 0.485 |
|  | *cnf1* | 2 (6) | 0 (0) | 2 (12.5) | 0.227 |
|  | *tosA* | 1 (3) | 0 (0) | 1 (6.3) | 0.485 |
|  | *tosB* | 1 (3) | 0 (0) | 1 (6.3) | 0.485 |
| Iron acquisition | ***fecA*** | 22 (66.7) | 15 (88) | 7 (43.8) | **0.009** |
|  | ***fyuA*** | 18 (54.5) | 5 (29) | 13 (81.3) | **0.004** |
|  | *iutA* | 9 (27.3) | 5 (29) | 4 (25) | 0.543 |
|  | *fepA* | 29 (87.9) | 13 (76.5) | 16 (100) | 0.058 |
|  | *entB* | 29 (87.9) | 13 (76.5) | 16 (100) | 0.058 |
|  | *iroN* | 12 (36.4) | 8 (47) | 4 (25) | 0.170 |
|  | ***irp2*** | 18 (54.5) | 5 (29) | 13 (81.3) | **0.004** |
|  | *iucA* | 9 (27.3) | 5 (29) | 4 (25) | 0.543 |
|  | *iha* | 3 (9) | 1 (5.9) | 2 (12.5) | 0.477 |
| Autotransporters | *aida* | 19 (57.6) | 11 (64.7) | 8 (50) | 0.308 |
|  | *vat* | 1 (3) | 0 (0) | 1 (6.3) | 0.485 |
|  | *sat* | 3 (9) | 1 (5.9) | 2 (12.5) | 0.477 |
|  | *pic* | 2 (6) | 1 (5.9) | 1 (6.3) | 0.742 |
|  | *pic-like* | 3 (9) | 1 (5.9) | 2 (12.5) | 0.477 |
|  | *pssA* | 0 (0) | 0 (0) | 0 (0) | 1.0 |
|  | *boa* | 0 (0) | 0 (0) | 0 (0) | 1.0 |
|  | *hbp* | 2 (6) | 1 (5.9) | 1 (6.3) | 0.742 |
|  | *ag43* | 15 (45.5) | 5 (29) | 10 (62.5) | 0.059 |
| Capsule related | ***kspMTII*** | 6 (18) | 0 (0) | 6 (37.5) | **0.007** |

^1^studied genes, their functions are described in the main text;

^2^The prevalence rates of the virulence genes among commensal versus pathogenic isolates were compared by the Chi-squared test of independence or Fisher’s exact test. P <0.05.
